# Exploring the Role of Action Consequences in the Handle-Response Compatibility Effect

**DOI:** 10.3389/fnhum.2020.00286

**Published:** 2020-07-31

**Authors:** Elisa Scerrati, Stefania D’Ascenzo, Luisa Lugli, Cristina Iani, Sandro Rubichi, Roberto Nicoletti

**Affiliations:** ^1^Department of Education and Human Sciences, University of Modena and Reggio Emilia, Reggio Emilia, Italy; ^2^Department of Biomedical, Metabolic and Neural Sciences, University of Modena and Reggio Emilia, Reggio Emilia, Italy; ^3^Department of Philosophy and Communication, University of Bologna, Bologna, Italy; ^4^Department of Surgery, Medicine, Dentistry and Morphological Sciences With Interest in Transplant, Oncology and Regenerative Medicine, University of Modena and Reggio Emilia, Modena, Italy; ^5^Center for Neuroscience and Neurotechnology, University of Modena and Reggio Emilia, Modena, Italy

**Keywords:** handle-response compatibility, response-effect compatibility, common coding of intention and action, ideomotor theory, affordance

## Abstract

Previous research investigating handle-response compatibility effects with graspable objects used different categories of objects as stimuli, regardless of their specific, intrinsic characteristics. The current study explores whether different types of objects’ characteristics may elicit different types of spatial compatibility, that is, handle-response and response-effect compatibility as well as their potential interaction. In Experiment 1, objects having a graspable handle opposite to either a visible functional component (i.e., *handle-function objects*: a teapot) or a latent functional component (*handle-only objects*: a pitcher lacking the spout) were presented separately in different blocks. Both the handle and the goal-directed functional components of these objects were located on the horizontal axis. In Experiment 2, handle-only objects had a handle located on the horizontal axis and a latent functional component located on the vertical axis (e.g., a cup). In both experiments, participants were required to judge the material (plastic and metal) the object was made of. Results showed that the handle-response compatibility effect was sensitive to whether the actions consequences of object manipulation took place on the horizontal rather than on the vertical axis.

## Introduction

The handle-response (H-R) compatibility effect (Iani et al., 2018; Pellicano et al., 2020), also known as handle-alignment (Bub et al., 2018) and object-based correspondence effect (Lien et al., 2013), refers to the finding of faster and more accurate responses when the position of an object’s graspable component (i.e., the handle) and the stimulus’ required response lay on the same side compared to when they lay on opposite sides.

Evidence in favor of the H-R compatibility effect was initially provided by Tucker and Ellis (1998) who showed that judging the upright or inverted position of depicted graspable objects was influenced by the orientation of the object’s handle. That is, responses were faster when the position of the handle and the responding hand were spatially aligned as compared to when they were not. This result was replicated across different tasks (e.g., Tipper et al., 2006; Saccone et al., 2016), stimuli (e.g., Pellicano et al., 2010; Pappas, 2014; Iani et al., 2018; Scerrati et al., 2019, 2020), populations (e.g., Dekker and Mareschal, 2013), response devices (e.g., Bub and Masson, 2010), and response modes (e.g., Phillips and Ward, 2002; Cho and Proctor, 2010; Proctor et al., 2017; Bub et al., 2018; for a review see Proctor and Miles, 2014; for a recent meta-analysis see Azaad et al., 2019).

The H-R compatibility effect has been argued to reflect the activation of action potentiation mechanisms driven by the affordances (Gibson, 1979) of objects (e.g., Tucker and Ellis, 1998; Tipper et al., 2006; Pellicano et al., 2010; Iani et al., 2011, 2018; Pappas, 2014; Saccone et al., 2016). That is, perceiving action-relevant visual features of objects such as a cup’s handle might trigger the affordance for grasping it with the left or right hand, which in turn generates a left or right code consisting of the activation of limb-specific motor patterns. This assumption, known as the *action-potentiation* or *affordance hypothesis*, has, however, been challenged by research showing null (Symes et al., 2005; Tipper et al., 2006; Loach et al., 2008; Bub and Masson, 2010; Kostov and Janyan, 2012; Cho and Proctor, 2013; Song et al., 2014; Yu et al., 2014; Saccone et al., 2016; Pellicano et al., 2020) and/or reversed (Pellicano et al., 2010, 2020; Cho and Proctor, 2011, 2013; Yu et al., 2014; Kostov and Janyan, 2015; Proctor et al., 2017) H-R compatibility effects. For instance, several studies failed to find H–R compatibility effects when participants made color judgments about a wide range of graspable objects (Symes et al., 2005; Tipper et al., 2006; Loach et al., 2008; Bub and Masson, 2010; Cho and Proctor, 2011; Saccone et al., 2016), whereas a few others obtained null effects with shape and horizontal/diagonal judgments of door-handles (Cho and Proctor, 2013), length and size judgments of passive (i.e., switched off) torches (Pellicano et al., 2020), and even with upright/inverted orientation judgments of different types of stimuli (Cho and Proctor, 2011; Kostov and Janyan, 2012; Song et al., 2014; Yu et al., 2014). Similarly, reversed H-R compatibility effects were found when participants discriminated both the color (Pellicano et al., 2010; Cho and Proctor, 2013) and the upright/inverted orientation (Kostov and Janyan, 2015; Proctor et al., 2017) of graspable objects, as well as when they categorized stimuli as being artifacts or natural kinds (Yu et al., 2014), judged the length and size of active (i.e., switched on) torches (Pellicano et al., 2020) or the color and orientation of teapot stimuli that featured spouts protruding to the opposite side of the handle (Cho and Proctor, 2011). All known null and reversed H-R compatibility effects are summarized in Table 1.

**TABLE 1:** Prior known tests of the Handle–Response (H–R) compatibility effect showing null and/or reversed effects.

Source, Study	*N*	Stimuli	Task	H–R compatibility effect
Bub and Masson (2010)				
2	54	Beer mugs/Teapots	Color-judgement	Null
Cho and Proctor (2011)				
4	32	Door-handles	Color/Orientation judgement	Null
Cho and Proctor (2013)				
1	40	Door-handles	Shape-judgement	Null
6	20	Door-handles	Diagonal orientation judgement	Null
Kostov and Janyan (2012)				
1	37	Frying pans, Jugs, Saucepans	Orientation judgement	Null
Loach et al. (2008)				
2	20	Door-handles	Color-judgement	Null
Pellicano et al. (2020)				
1	20	Passive Torches	Length judgement	Null
2	20	Passive Torches	Length judgement	Null
3	20	Passive Torches	Size judgement	Null
Saccone et al. (2016)				
2	25	Mostly common elongated tools	Color-judgement	Null
Song et al. (2014)				
1	32	Passive Torches	Orientation judgement	Null
Symes et al. (2005)				
3	33	Mostly common elongated tools	Color-judgement	Null
Tipper et al. (2006)				
1	32	Door-handles	Color-judgement	Null
Yu et al. (2014)				
2A	30	Mostly common elongated tools	Orientation judgement	Null
2B	30	Mostly common elongated tools	Orientation judgement	Null
3A	30	Mostly common elongated tools	Orientation judgement	Null
Cho and Proctor (2011)				
1	64	Teapots	Color/Orientation judgement	Reversed
Cho and Proctor (2013)				
2	80	Door-handles	Color-judgement	Reversed
3	20	Door-handles	Color-judgement	Reversed
4	60	Door-handles	Color-judgement	Reversed
5	20	Door-handles	Color-judgement	Reversed
Loach et al. (2008)				
1	20	Door-handles	Texture judgement	Reversed
Kostov and Janyan (2015)				
2	58	Frying pans, saucepans bowls/plates	Orientation judgement	Reversed
3	51	Frying pans, saucepans bowls/plates	Orientation judgement	Reversed
Kourtis and Vingerhoets (2015)				
	29	Mostly common elongated tools	S-R compatibility	Reversed
Pellicano et al. (2010)				
1	20	Torches	Color judgement	Reversed
Pellicano et al. (2020)				
2	20	Active Torches	Length judgement	Reversed
3	20	Active Torches	Size judgement	Reversed
Proctor et al. (2017)				
1	20	Frying pans	Orientation judgement	Reversed
Yu et al. (2014)				
1A	32	Mostly common elongated tools	Artifact/Natural	Reversed
1B	32	Mostly common elongated tools	Artifact/Natural	Reversed
1C	32	Mostly common elongated tools	Artifact/Natural	Reversed

This consistent body of evidence led several authors to raise questions about the *affordance hypothesis*. In particular, it has been argued that the H-R compatibility effect may not involve the activation of the motor system (e.g., Cho and Proctor, 2010, 2011, 2013; Proctor et al., 2017; see Proctor and Miles, 2014 for a review). Rather, according to supporters of the *location coding* and the *feature-asymmetry* accounts (Cho and Proctor, 2010, 2013; Song et al., 2014), the graspable component of an object creates visual asymmetries within the stimulus display that become perceptually salient to the observer. The location of these salient portions generates a left or right spatial code that may or may not correspond with the spatial code for the response (see also Lien et al., 2013; Lien et al., 2014; Song et al., 2014). As other spatial compatibility effects such as the Simon effect (Simon and Rudell, 1967; Simon and Small, 1969; Simon, 1990; for recent investigations of the Simon effect see Scerrati et al., 2017; D’Ascenzo et al., 2018), the H-R compatibility effect would, thus, be due to a dimensional overlap (Kornblum et al., 1990; Kornblum and Lee, 1995; Kornblum and Stevens, 2002) between stimuli and response spatial features.

However, it is interesting noting that several studies obtaining null and/or reversed H-R compatibility effects employed the images of many different common elongated tools as stimuli (e.g., ladles, hammers, screwdrivers, and strainers, etc.), which were displayed horizontally on the monitor (see Table 1 for an overview). Therefore, such tools had the functional component laying on the opposite side than the handle component, both on the horizontal axis. Other studies among those observing null/reversed H-R compatibility effects used images of teapots (e.g., Symes et al., 2005; Bub and Masson, 2010; Cho and Proctor, 2011; Yu et al., 2014; Saccone et al., 2016), jugs (e.g., Symes et al., 2005; Kostov and Janyan, 2012; Yu et al., 2014; Saccone et al., 2016) and watering cans (e.g., Yu et al., 2014; Saccone et al., 2016), which likely drove participants to anticipate the effects of their use unfolding on the opposite side than the handle. Still, other studies reporting null/reversed H-R compatibility effects used images of torches as stimuli (e.g., Pellicano et al., 2010, 2020; Song et al., 2014), which likely induced participants to expect consequences of their switching on/off appearing on the side opposite to the handle. Perhaps less obvious, but also those studies using a door-handle as the target stimulus (e.g., Tipper et al., 2006; Loach et al., 2008; Cho and Proctor, 2011, 2013) may have induced participants to anticipate the typical downward rotation necessary for operating any door-handle, which may be seen as moving the door-handle to the side opposite to the tip of the handle on the horizontal axis. Therefore, as illustrated by the examples above, the effect of a further, potential moderator of the H-R compatibility effect may be responsible if not for all, at least for a part of the null and reversed H-R compatibility effects reported in Table 1. Such moderator is the effect of the representation of intentions and actions that people would likely anticipate when seeing an object.

Importantly, Hommel (1993) demonstrated that the intention to produce an effect (turn on a light) on a side opposite to that of responding generates a response facilitation when the stimulus and effect sides overlap. More specifically, participants were faster at responding to the pitch of target tones through left- and right-hand keypresses when these tones were delivered through a loudspeaker located on the same side as the light lighting up as a result of the response. Further work on the impact of action anticipation on compatibility effects led to the discovery of a response-effect compatibility, which was initially investigated by Kunde (2001) who had participants discriminating the color of a centrally presented circular dot by pressing one of four horizontally aligned keys (one for each of four possible colors). As a result of the key press, one of four horizontally aligned boxes presented on the screen above the keys became white. Results showed faster responses when the position of the box that became white and the response key were compatible (one above the other) rather than incompatible (one located two positions adjacent to the other). Furthermore, Ansorge (2002) presented participants with a central stimulus, either the letter H or T, and asked them to move it toward the right or the left on the screen by pressing one of the two mouse keys. The author found that responses were faster when people had to press a key located in the same direction as the intended movement. In addition, Tagliabue et al. (2006) asked participants to drive a car in the context of virtual reality and to take a right or left turn on the basis of the color of two lateralized traffic lights. Participants had to push a joystick either toward the lit-up traffic light or in the opposite direction depending on whether the light was green or red. In the Coordinated Information condition, the car took the right turn if people push their joystick to the right and the left turn if people push their joystick to the left. In the uncoordinated information condition, the car took the right turn if people push their joystick to the left and the left turn if people push their joystick to the right. The authors found faster and more accurate responses in the Coordinated rather than Uncoordinated Information condition indicating that compatibility between the intended action effect (turn left/right) and the response (push the joystick left/right) matters.

The finding of faster and more accurate responses when the intended action effect of a stimulus (i.e., the effect it produces) and the response it requires share the same location in space, compared to when they do not, suggests that the representations of actions also contain the effects they produce. This assumption known as the *common coding hypothesis of intention and action* (Prinz, 1990, 1997; Jeannerod, 1999; Elsner and Hommel, 2001; see also Greenwald, 1970 and James, 2013 for a previous version of the common coding hypothesis known as ideomotor theory) entails that people would inevitably anticipate the cognitive representation of actions’ consequences. It has been proposed that one potential anatomical correlate of predictive motor control is the cerebellum (Ito, 1970). The cerebellum instantiates neural mechanisms called internal models (i.e., forward and inverse models) that capture the causal relationship between actions and their consequences (Wolpert et al., 1998; Kawato, 1999). In other words, seeing an object might either lead to the activation of the effects produced by actions with the object or to the representation of the intention to produce those effects (Hommel, 1993, 1996; Elsner and Hommel, 2001; Kunde, 2001; Ansorge, 2002; Pfister et al., 2014). Thus, the effects act as stimuli that, although being task irrelevant, are processed before response initiation (Greenwald, 1970; James, 2013; see Shin et al., 2010 for a review).

Importantly, for the purpose of the present study, handle-response and response-effect compatibility have so far been addressed separately by different studies. The aim of the present study is to explore their concurrent effects in order to shed light on their potential interactions when participants process pictures of graspable objects.

## The Present Research

In the current study, we examined whether response-effect compatibility can influence handle-response compatibility. To this end, we manipulated the specific type of object presented to participants.

Some objects’ graspable components (i.e., the handles) are opposite to the intended action effects or consequences of their manipulation. Consider, for example, grasping a teapot in order to pour its content into a cup: people will likely grasp it with their dominant hand and pour its content in the opposite direction. Within this class of objects, some have a clearly visible or explicit goal-directed functional component (i.e., think, for example, to the spout of a teapot), whereas others have a latent or implicit goal-directed functional component (i.e., many milk pots on the market lack the spout, for example). We will refer to the former as handle-function objects and to the latter as handle-only objects. For both types of objects, the intended action effects or consequences of their manipulation are opposite to the side where the grasping movement they afford takes place. That is, both teapots and milk pots are grasped on one side, whereas the intended action effects of their use occur on the opposite side on the horizontal axis. Importantly, for some handle-only objects the intended action effects of their use occur on the vertical axis. For instance, grasping a cup in order to sip its content entails bringing it to the mouth through a vertical ascending movement of the arm. We tested whether these intrinsic differences of objects have an impact on handle-response compatibility. Indeed, Tipper et al. (2006) highlighted that “Subtle differences in the visual stimuli [can have] a dramatic effect” on the H–R compatibility effect (p. 496), and Yu et al. (2014) pointed out that “[…] certain stimuli may be more likely to prime actions” (p. 1868).

## Overview of the Experiments

In two experiments, we presented participants with objects having either a visible goal-directed functional component located on the horizontal axis (i.e., handle-function objects) or a latent goal-directed functional component (i.e., handle-only objects) located on the horizontal (Experiment 1) or the vertical axis (Experiment 2).

Importantly, as for handle-only objects having a latent goal-directed functional component located on the horizontal axis, there was little point in using stimuli from previous studies such as frying pans (Proctor et al., 2017), or saucepans (Kostov and Janyan, 2012, 2015), since these objects are more commonly used with the help of other tools (e.g., we often use a spatula or the likes to flip and serve the pancakes), and seldom have actions associated with them that clearly involve a horizontal axis. We, therefore, used objects the content of which can be poured.

Participants had to recognize the material each object was made of by pressing one of two keys on the keyboard. We were interested in the task being action-relevant since previous studies consistently showed that task characteristics may affect the H-R compatibility effect (e.g., Symes et al., 2005; Tipper et al., 2006; Loach et al., 2008; Pellicano et al., 2010; Saccone et al., 2016; see Azaad et al., 2019 for a discussion). As Yu et al. (2014) pointed out: “[…] the likelihood of observing action priming, either positive or negative, will be reduced when a task can be accomplished without considering the potential actions associated with a target object, as when judging the color of an object” (p. 1867). Thus, we chose a material discrimination task as different materials can influence the grasping movement required by an object giving rise to different actions with objects (for similar tasks see Weir et al., 1991; Fikes et al., 1994; Loach et al., 2008). For example, grasping a metal milk pot after it has been on the stove will probably require a careful movement because of the temperature of the object, whereas grasping a plastic measuring cup certainly will not.

According to the *action potentiation account*, handle-function objects, that is, objects with a graspable handle and a visible goal-directed functional component located on opposite sides, should elicit the H-R compatibility effect because of the orientation of the handle component, which is deemed to induce people to activate motor codes compatible with the side of the handle. However, according to the *common coding hypothesis*, these same objects should also elicit the response-effect compatibility because of the orientation of the clearly visible goal-directed functional component, which is assumed to induce people to expect effects occurring on the functional side of these objects. Therefore, we expected the two compatibility effects to offset each other, this resulting in a null spatial compatibility effect. Of course, a null spatial compatibility effect could also be due to handle-function objects generating opposing left/right spatial codes (induced by the protrusion of the spout and the protrusion of the handle) as claimed by the *location coding* account.

Conversely, handle-only objects with a graspable handle and a latent goal-directed functional component located horizontally on opposite sides could provide support either in favor of a combined influence of action potentiation and common-coding mechanisms or they could generate results in line with a *location-coding* account. According to the *location coding* account (Cho and Proctor, 2010; see also Cho and Proctor, 2013; Song et al., 2014) these stimuli should elicit the H-R compatibility effect because of the available spatially relevant protrusion (i.e., the handle) on one side. Contrary, according to the combined *action potentiation and common coding* hypotheses (Prinz, 1990, 1997; Jeannerod, 1999; Elsner and Hommel, 2001), these objects should bring about a null effect because of a H-R compatibility effect generated by the action-relevant component (i.e., the handle), and a response-effect compatibility effect generated by the latent goal-directed functional component opposite to the handle. Indeed, according to the *common coding* hypothesis, even if the goal-directed component is latent, it may nevertheless induce people to expect action to occur on the opposite side than the handle’s side because intentions, actions and their effects are represented through a common coding and effects act as a source of stimulation although being non-salient and task irrelevant.

Importantly, in Experiment 2 we replaced handle-only objects the intended action effects of which occur on the horizontal axis (e.g., a pitcher lacking the spout) with those for which the same effects occur on the vertical axis (e.g., a cup). Our aim was to test whether different actions’ consequences of object manipulation (i.e., taking place on the vertical rather than the horizontal axis) can influence H-R and response-effect compatibility effects with otherwise same sized and similarly shaped objects. According to the combined *action potentiation* and *common coding* hypotheses, handle-only objects in Experiment 2 should show an effect of handle-response compatibility and no effect of response-effect compatibility as these objects lack a goal-directed functional component, either visible or latent, on the horizontal axis and the intended action effects of their use occur on the vertical axis. We are aware that the same result is predicted by the *location coding* account, since the handle is the only protrusion available within these objects. However, the rationale underlying our manipulation rests on the comparison between results concerning handle-only objects across Experiments 1 and 2. Therefore, a null H-R compatibility effect for handle-only objects in Experiment 1 together with a significant and positive H-R compatibility effect for handle-only objects in Experiment 2 will be taken as evidence in favor of a combined influence of action potentiation and common coding mechanisms and against a *location coding* account. See Table 2 for a summary of the predicitons.

**TABLE 2:** Summary table of the expected Handle-Response (HR) and Response-Effect (RE) compatibility effects for both handle-function and handle-only objects according to the action potentiation, common coding and location coding accounts.

Account	Handle-function objects			Handle-only objects from Exp. 1			Handle-only objects from Exp. 2		
	H-R	R-E	Resulting effect	H-R	R-E	Resulting effect	H-R	R-E	Resulting effect
Action potentiation	✓	—	Null	✓	—	Null	✓	—	H-R
Common coding	—	✓		—	✓		—	—	—
	**Visual salience**			**Visual salience**			**Visual salience**		
Location coding	Handle	Spout	Null	Handle	—	H-R	Handle	—	H-R

## Experiment 1

### Method

#### Participants

We calculated the sample size required to achieve 80% power to detect a significant interaction between handle-response compatibility (compatible vs. incompatible) and type of object (handle-function vs. handle- only) with the G^∗^power 3.1 (Faul et al., 2007) software. With an effect size *f* = 0.25 (Cohen, 1988), the power calculation yielded a recommended sample size of at least 24 participants^1^.

Twenty-seven students from the University of Bologna (16 females, four left-handed, *M*_age_ = 21.3, SD_age_ = 1.9) served as participants. All reported normal or corrected-to-normal vision and were naïve as to the purpose of the experiment. This and the following experiments were conducted in accordance with the ethical standards laid down in the Declaration of Helsinki and fulfilled the ethical standard procedure recommended by the Italian Association of Psychology (AIP). All procedures were approved by the ethics committee of the University of Bologna. All participants gave their written informed consent to participate to the study.

#### Apparatus, Stimuli, and Procedure

The experiment was conducted in a quiet room, where the light was dimmed. Stimuli were presented on a Dell 22-inch (56 cm) video monitor (refresh rate: 60 Hz; resolution: 1280 × 800 pixels) on a white background. The viewing distance was 60 cm. Stimuli presentation and responses collection were controlled by E-Prime Professional v2.0 software^2^. The stimuli consisted of digital photographs of four different domestic objects (measuring cup, pitcher, teapot, and watering can) selected from public-domain images available on the Internet. Selection was made by considering the type of object (handle-function: explicit handle and function, both on the horizontal axis; handle-only: explicit handle and implicit function, both on the horizontal axis) and the object’s material (metal vs. plastic). Each picture was adjusted to an average luminance value, rendered in grayscale and resized to 4.5 cm × 4.5 cm (subtending a maximum visual angle of 4.3° × 4.3°). Each stimulus was centrally displayed according to the length and width of the entire object. Objects measured 2.25 cm (2.15° of visual angle) from the central fixation cross to their left/right end(s).

**FIGURE 1:**
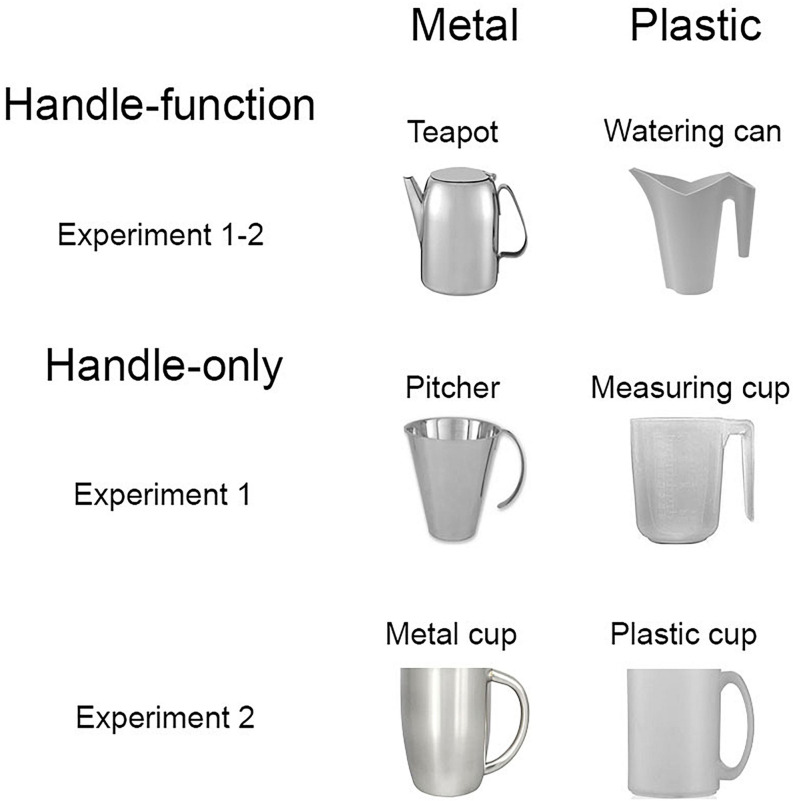
Handle-function (i.e., upper panel), and handle-only (middle and lower panel) objects made of metal (leftmost panel) and plastic (rightmost panel) used in the two experiments. All objects are depicted with the handle on the right. Elements are not drawn to scale.

In order to prevent an effect of salience of the handle or goal-directed component, both components of the handle-function objects were of the same size (on the importance of salience for spatial compatibility effects see Lamberts et al., 1992; Cho and Proctor, 2010, 2013; Lien et al., 2014; Proctor et al., 2017). Objects could be presented with a leftward- or rightward-oriented handle. For each type of object (handle-function; handle-only) there was one metal and one plastic object (see Figure 1 for details).

Participants were instructed to respond according to the material (metal vs. plastic) of the object. Responses were executed by pressing the left (i.e., “Alt”) or right (i.e., “Ctrl”) response keys on an Italian QWERTY keyboard with the left and right index fingers, respectively.

At the beginning of the experiment, the fixation cross appeared in the middle of the screen for 1000 ms. Then, the stimulus was centrally displayed according to the length and width of the entire object until a response occurred. Half of the participants were instructed to respond as quickly and accurately as possible to the plastic stimulus by pressing the left response key and to the metal stimulus by pressing the right response key. The other half experienced the opposite mapping rule. In handle-response compatible trials, the handle position and the key for the correct response were on the same side (e.g., handle on the right, correct response on the right), whereas in handle-response incompatible trials, the handle was located on the opposite side with respect to the position of the correct response (e.g., handle on the left, correct response on the right).

Participants underwent a training phase consisting of 16 practice trials. Following the training, there were 4 experimental blocks of 96 trials each, for a total of 384 experimental trials.

To rule out potential carry-over effects due to a mixed presentation of the type of object (i.e., handle-function, handle-only), the two types of objects were presented separately in different blocks. A pilot study from our lab mixing handle-function and handle-only objects within the same block yielded reversed H-R compatibility effects for handle-only objects likely due to a carry-over of handle-function on handle-only objects (see Scerrati and D’Ascenzo, 2018). Similarly, Pellicano et al. (2010) found a reversed H-R compatibility effect when they presented different types of objects (i.e., active/passive torches) in different blocks and counterbalanced the order of blocks across participants. That is, some participants viewed blocks of active torches first, whereas others viewed blocks of passive torches first. When presented first, active torches may have biased participants’ attention toward the functional side of the torches thus inverting the effect for both types of objects. Active/passive torches can be assimilated to our handle-function/handle-only objects, respectively. Therefore, in order to avoid a potential influence of handle-function on handle-only objects, in the present experiments the latter were presented in the first two blocks, whereas the former were presented in the last two blocks.

An equal number of trials was provided for each combination of the following variables: stimulus material (metal vs. plastic), type of object (handle-only vs. handle-function) and handle-response compatibility (compatible vs. incompatible).

### Analysis

Practice trials, reaction times (RTs) faster or slower than 2 SD from the participant’s mean (2.7% of the total trials) and errors (3.4% of the total trials) were excluded from the analysis on RTs. For simplicity and in line with previous studies (e.g., Iani et al., 2018), data were collapsed based on the spatial compatibility between handle orientation and response position (handle-response compatible vs. handle-response incompatible trials).

Repeated measures ANOVAs were conducted on correct RTs and arcsine-transformed error rates (ERs) with *Handle-Response Compatibility* (compatible vs. incompatible) and *Type of Object* (handle-function vs. handle-only), as within-subjects’ factors. The Huynh–Feldt correction was used when appropriate. The effect size was estimated by calculating the partial eta squared statistic (η_p_^2^).

### Results

#### Reaction Times

The analysis showed a significant main effect of the *Type of Object*, *F*(1,26) = 11.538, MSE = 1508.093, *p* = 0.002, η_p_^2^ = 0.307, with faster responses for handle-function (474 ms) compared to handle-only (499 ms) objects.

**FIGURE 2:**
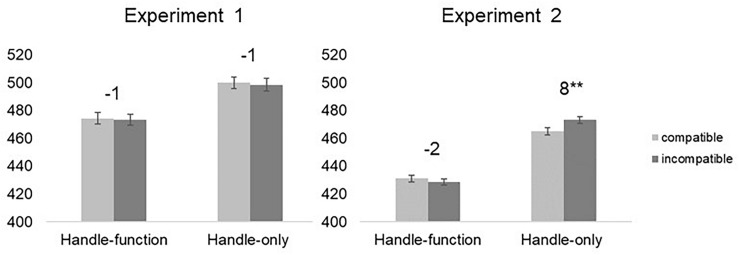
Mean reaction times (RTs) as a function of *Handle–Response Compatibility* (compatible vs. incompatible) and *Type of Object* (handle-function vs. handle-only) for Experiment 1 (leftmost panel) and Experiment 2 (rightmost panel). Error bars indicate standard errors of the mean adjusted for within-participants designs (Loftus and Masson, 1994). The magnitude of the H-R compatibility effect (computed by subtracting mean RT for compatible trials from that for the incompatible trials) is reported on top of bars, separately for each type of object. Asterisks denote significant H-R compatibility effects (^∗∗^*p* < 0.01).

Neither the main effect of the *Handle-Response Compatibility*, nor its interaction with the *Type of Object* were significant [*F*(1,26) = 0.267, *p* = 0.609, η_p_^2^ = 0.010; *F*(1,26) = 0.005, *p* = 0.942, η_p_^2^ < 0.001, respectively]. In particular, the handle-response compatibility effect was not significant for both handle-function and handle-only objects (−1 ms for both objects, *t*(26) = −0.274, *p* = 0.786 and *t*(26) = −0.523, *p* = 0.605, respectively) (see Figure 2).

#### Error Rates

The analysis showed neither significant main effects nor interaction, all *F* < 1.

### Discussion

Results from Experiment 1 showed that participants were faster to respond to handle-function compared to handle-only objects. This result might be due to the fact that a handle-function object explicitly suggests where the types of interactions it affords and the types of actions it allows to accomplish are going to take place, whereas a handle-only object only suggests the former without any explicit hint to the latter. Such a difference might delay the processing of handle-only objects thus incurring a cost. In other words, participants may linger over the functional side of handle-only objects in search for the latent goal-directed component.

Importantly, Experiment 1 showed that the type of object did not modulate the H-R compatibility effect, that is, both types of object yielded null H-R compatibility effects. The null effect for handle-function objects supports the combined *action potentiation* and *common coding* explanations as well as the *location coding* account. According to the former, the handle and the functional component of objects may yield to the co-occurrence of effects of handle-response and response-effect compatibility, respectively, which offset each other. According to the latter, competing left/right spatial codes could have been generated by the protrusion of the handle and the protrusion of the spout.

Interestingly, the null effect for handle-only objects may be taken as preliminary evidence in favor of the *common coding hypothesis of intention and action* (Prinz, 1997; Jeannerod, 1999; Elsner and Hommel, 2001). That is, despite being latent the goal-directed functional component of these stimuli likely induced people to anticipate the effects of an action unfolding on the functional side of the object, thus producing an effect of response-effect compatibility. Such effect combined with the H-R compatibility effect, brought about by the handle component, resulted in a null spatial compatibility effect. In contrast, according to the *location coding* account (Cho and Proctor, 2010; see also Cho and Proctor, 2013; Song et al., 2014) the available salient protrusion of these stimuli (i.e., the handle) should have elicited an H–R compatibility effect.

However, the conclusion that handle-only objects yielded a null effect because of the simultaneous effect of handle-response and response-effect compatibility calls for further test. To this end, in Experiment 2 handle-only objects were replaced with new ones. The new objects were of the same size and similar shape as those used in Experiment 1. Importantly, the effects of their use now occurred on the vertical rather than the horizontal axis. These new handle-only objects were expected to yield an effect of handle-response compatibility and no effect of response-effect compatibility as they lack a goal-directed functional component, either visible or latent, on the horizontal axis.

## Experiment 2

Experiment 1 showed a null handle-response compatibility effect for both handle-function and handle-only objects. This result provides somewhat preliminary evidence in favor of the *common coding hypothesis of intention and action* (Prinz, 1997; Jeannerod, 1999; Elsner and Hommel, 2001), which posits that effects act as stimuli, and intention, action and their effects share a common coding.

However, the absence of an effect concerning handle-only objects is partial evidence in favor of the *common coding hypothesis*. Experiment 2 sought to find more robust evidence by introducing handle-only objects the manipulation of which produces effects on the vertical rather than the horizontal axis.

If the null effect concerning handle-only objects observed in Experiment 1 was due to response-effect compatibility, as predicted by the *common coding hypothesis*, we expect to find a H-R compatibility effect for handle-only objects that are of the same size and similar shape as those used in Experiment 1 but the intended action effects of which occur on the vertical rather than on the horizontal axis. Indeed, according to the *common coding hypothesis*, no effect of response-effect compatibility that could counteract the effect of handle-response compatibility should emerge in this case, as these objects lack a goal-directed functional component, either visible or latent, on the horizontal axis. In contrast, handle-function objects were expected to show the null H-R compatibility effect observed in the previous experiment.

### Method

#### Participants

Twenty-seven new students from the University of Bologna (14 females, one left-handed, *M*_age_ = 23.3, SD_age_ = 7.43) served as participants. All reported to have normal or corrected-to-normal vision and were naïve as to the purpose of the experiment.

#### Apparatus, Stimuli, and Procedure

Apparatus and procedure were the same as in Experiment 1. Two new stimuli were selected as handle-only objects^3^ (see Figure 1, lower panel). In particular, a cup made of metal and a cup made of plastic, each with a visible handle component on the horizontal axis and a latent function on the vertical axis, were chosen.

As in Experiment 1 these objects were centered on screen according to the length and width of the entire object. In keeping with Experiment 1, they were presented in the first two experimental blocks in order to avoid potential carry over effects of handle-function objects (Pellicano et al., 2010; Scerrati and D’Ascenzo, 2018).

### Analysis

Practice trials, RTs faster or slower than 2 SD from the participant’s mean (3.8% of the total trials) and errors (4.1% of the total trials) were excluded from the analyses. Correct RTs and ERs were analyzed as in the previous experiments.

### Results

#### Reaction Times

The analysis showed a significant main effect of the *Type of Object*, *F*(1,26) = 94.585, *MSE* = 442.573, *p* < 0.001, η_p_^2^ = 0.784, indicating that responses were faster with handle-function (430 ms) than with handle-only (469 ms) objects. The main effect of *Handle-Response Compatibility* was not significant, *F*(1,26) = 2.363, MSE = 91.332, *p* = 0.136, η_p_^2^ = 0.083.

Crucially, the interaction of the *Handle–Response Compatibility* and the *Type of Object* was significant, *F*(1,26) = 6.859, MSE = 112.861, *p* = 0.015, η_p_^2^ = 0.209. To illustrate, we detected a non-significant handle-response compatibility effect of −2 ms for handle-function objects and a significant handle-response compatibility effect of 8 ms for handle-only objects [*t*(26) = −0.867, *p* = 0.394 and *t*(26) = 3.177, *p* = 0.004, Cohen’s *d* = 0.13, respectively]^4^ (see Figure 2, right panel).

Importantly, an independent sample t test was performed in order to compare the magnitude of the H–R compatibility effect concerning handle-only objects across Experiments 1 and 2. Results demonstrated that the two effects differed significantly, *t*(26) = −2.485, *p* = 0.016.

#### Error Rates

The analysis showed a significant main effect of the *Type of Object*, *F*(1,26) = 24.350, MSE = 0.007, *p* < 0.001, η_p_^2^ = 0.484, indicating that participants made less errors with handle-function (2.5%) than handle-only (5.4%) objects. No other main effect or interaction was significant, *F_s_* < 2.239, *p* > 0.147.

### Additional Analyses

One might argue that the observed pattern of results could be affected by objects differing on other dimensions than the one we hypothesized (i.e., familiarity of objects, their frequency of use, etc.). To rule out this possibility, we ran multiple paired sample *t*-tests separately for each Experiment (1, 2) with the purpose of comparing mean response times obtained for each object identity (e.g., teapot, watering can) within each type of object (i.e., handle-function; handle-only) and compatibility condition (i.e., H-R compatible, H-R incompatible). Crucially, there were no significant differences between object identities within neither handle-function nor handle-only objects (see Table 3). Therefore, we conclude that our results were not affected by confounding factors between stimuli.

**TABLE 3:** Results from multiple paired sample *t*-tests performed separately for each Experiment (1, 2), type of object pair (handle-function, handle-only), and compatibility condition (H-R compatible, H-R incompatible).

Type of object	Objects pair	H-R compatible	*T*-test	H-R incompatible	*T*-test
**Experiment 1**					
Handle-function	Teapot	475 ms	*t*(26) = 0.459, *p* = 0.650	472 ms	*t*(26) = 0.519, *p* = 0.608
	Watering can	473 ms		474 ms	
Handle-only	Measuring cup	499 ms	*t*(26) = 0.297, *p* = 0.769	499 ms	*t*(26) = 0.039, *p* = 0.969
	Pitcher	500 ms		498 ms	
**Experiment 2**					
Handle-function	Teapot	431 ms	*t*(26) = 0.845, *p* = 0.406	426 ms	*t*(26) = 1.918, *p* = 0.066
	Watering can	436 ms		435 ms	
Handle-only	Metal cup	468 ms	*t*(26) = 0.424, *p* = 0.675	471 ms	*t*(26) = 1.312, *p* = 0.201
	Plastic cup	466 ms		478 ms	

Furthermore, since it is very frequent to observe a learning effect with last blocks showing faster response times than first blocks we tested whether the H-R compatibility effect differs between the first and the second block for both handle-only and handle-function objects, separately for each Experiment. As for Experiment 1, handle-only (horizontal) objects show a negative and non-significant H-R compatibility effect of −2.6 ms in block 1, *t*(26) = −0.614, *p* = 0.545 and a negative and non-significant H-R compatibility effect of −0.4 ms in block 2, *t*(26) = −1.113, *p* = 0.911. The two effects did not differ from each other, *t*(26) = −0.428, *p* = 0.672. Similarly, handle-function objects show a negative and non-significant H–R compatibility effect of −3.1 ms in block 3, *t*(26) = −0.583, *p* = 0.565 and a non-significant H–R compatibility effect of 1 ms in block 4, *t*(26) = 0.223, *p* = 0.825. The two effects did not differ from each other, *t*(26) = −0.829, *p* = 0.414. As for Experiment 2, handle-only (vertical) objects show a positive and significant H–R compatibility effect of 8.9 ms in block 1, *t*(26) = 2.424, *p* = 0.023 and a positive and significant H–R compatibility effect of 7.04 ms in block 2, *t*(26) = 2.305, *p* = 0.029. The two effects did not differ from each other, *t*(26) = −0.459, *p* = 0.650. Conversely, handle-function objects show a negative and non-significant H-R compatibility effect of −5 ms in block 3, *t*(26) = −1.413, *p* = 0.170 and a negative and non-significant H-R compatibility effect of −1.6 ms in block 4, *t*(26) = −0.442, *p* = 0.662. The two effects did not differ from each other, *t*(26) = −0.880, *p* = 0.387. Given these findings we can safely conclude that no learning effects may be responsible for the observed pattern of results in neither experiment.

### Discussion

Overall, as already observed in the previous experiment, results from Experiment 2 showed that participants were faster (and this time also more accurate) to respond to handle-function objects compared to handle-only objects. As discussed above this result might depend on the specific characteristics of the type of object (handle-function vs. handle-only). A possible alternative explanation is that the material of the two handle-function objects may have resulted easier to discriminate as these objects (i.e., a teapot and a watering can) are more different from each other than the two handle-only objects (i.e., two cups of different materials). However, given that the same result was obtained in Experiment 1, where handle-only objects were a pitcher and a measuring cup and as such easily distinguishable from one another, we believe this alternative explanation to be unlikely.

Critically, in Experiment 2 the H-R compatibility effect was modulated by the type of object. That is, consistent with the previous experiment, handle-function objects yielded to a null effect of handle-response compatibility. This result further suggests that a visible handle and a visible functional component may elicit the handle-response and response-effect compatibility, respectively.

Crucially, handle-only objects brought about an effect of handle-response compatibility. In line with the predictions from the *common coding hypothesis* no influence of response-effect compatibility that could yield to a null handle-response compatibility effect emerged with handle-only objects the intended action effects of which occur on the vertical axis.

## General Discussion

Previous research investigating H-R compatibility effects with graspable objects used different categories of objects as stimuli, without distinguishing between them. We tested whether different types of objects’ characteristics may elicit different types of spatial compatibility effects by manipulating the location where the intended action effects of an object take place (horizontal *versus* vertical axis). Results demonstrate that whether the object is a handle-function object (e.g., teapot) with a visible handle and a visible functional component (e.g., spout) located on opposite sides on the horizontal axis or a handle-only object with a visible handle located on the horizontal axis and a latent functional component located either on the horizontal (e.g., pitcher lacking the spout) or on the vertical axis (e.g., cup) influences the H-R compatibility effect. Indeed, in both experiments handle-function objects (e.g., teapot) gave rise to a null H-R compatibility effect. Handle-only objects with action effects occurring on the horizontal axis (e.g., pitcher lacking the spout) elicited a null H-R compatibility effect in Experiment 1. Conversely, handle-only objects with action effects occurring on the vertical axis (e.g., cup) led to a H-R compatibility effect in Experiment 2.

The null H–R compatibility effect concerning handle-only objects observed in Experiment 1 may be taken as preliminary evidence in favor of the *common coding hypothesis of intention and action* (Prinz, 1997; Jeannerod, 1999; Elsner and Hommel, 2001) according to which the intended action effects of objects act as a source of stimulation producing response-effect compatibility even when the functional component of the object is latent.

It might be argued that the null effect concerning handle-only objects found in Experiment 1 rests on centering the entire object on screen rather than its base. Indeed, this object centering method implies that most image pixels appear contralateral to the object’s handle, thus weakening the spatial code elicited by the handle (e.g., Proctor et al., 2017). However, in Experiment 2, when same-sized handle-only objects were used and the same centering method was applied, an H-R compatibility effect emerged for handle-only objects the intended action effects of which occur on the vertical axis. Therefore, failing to observe an H-R compatibility effect for handle-only objects in Experiment 1 is unlikely to depend on the way objects were centered on screen. Rather, our results showed that the H-R compatibility effect was affected by the location of the functional component of the displayed object, and specifically, on whether it was on the horizontal rather than on the vertical axis. That is, an anticipatory effect of response effect compatibility likely occurred when the either visible or latent functional component of the object was located on the horizontal axis inducing people to anticipate actions consequences on the handle’s opposite side. It is worth emphasizing that these results suggest that intentions to interact with objects may direct attention to the action effects or consequences of the object manipulation even though actions consequences are neither relevant for the purposes of the task, nor perceptually salient (Ansorge, 2002; see also Jax and Buxbaum, 2010 for similar results with more naturalistic experimental settings). Therefore, it is likely that neural anticipatory mechanisms driven by the cerebellar internal models of the environment mimic the input-output properties of our own body and other objects (Wolpert et al., 1998; Kawato, 1999; see also Nowak et al., 2007; Imamizu and Kawato, 2009). Our finding of a response-effect compatibility effect further extends results from Pellicano et al. (2017) by showing that a response code relative to the functional side of the objects was created even if no action was suggested in the visual scene.

It is worth noting that our results on handle-only objects with action effects occurring on the vertical axis (i.e., cups) differ from previous findings reported by Bub and Masson (2010); Experiment 2) who failed to find an H-R compatibility effect for objects similar to ours (i.e., beer mug stimuli) when participants responded with keypresses. However, we would like to point out that these authors used color (blue, green) as the relevant cue, that is, participants were required to discriminate the color of beer mugs. It is well known that color judgments are less sensitive to affordance perception as demonstrated by a number of results available in the literature (e.g., Symes et al., 2005; Tipper et al., 2006; Loach et al., 2008; Pellicano et al., 2010; Saccone et al., 2016; see Table 1 for an overview; see Azaad et al., 2019 for a discussion).

Overall, by focusing on the investigation of potential, additional moderators of the H-R compatibility effect, this study bears new, underrated evidence on the interaction between handle-response and response-effect compatibility when processing graspable objects. Indeed, to the best of our knowledge no distinction has ever been made as to whether the intended effects of objects’ manipulation occur on the horizontal rather than the vertical axis when investigating the handle-response compatibility effect. We provided evidence suggesting that this aspect may be crucial and believe future studies should take it into account when selecting the stimulus set.

On a more general level, our experimental results may be informative for clinical studies reporting impaired perception of affordances for patients with schizophrenia. For example, Delerue and Boucart (2012) showed that these patients’ visual explorations of graspable objects favored the goal-directed functional component of tools. However, rather than impaired perception of affordance, patients with schizophrenia may be exploring the functional side of tools as a consequence of a loss of ease in their actions due to a disintegration of automatic practices that demand them to think deliberately about each action before performing it (Sevos et al., 2016). Therefore, it would be intriguing to manipulate the axis of visible/latent goal-directed components of tools when studying action anticipation and affordance perception in these patients. Likewise, we believe that our study may also have some clinical relevance when investigating affordance perception in brain-damaged patients with motor apraxia, who struggle at coordinating distal movements (Osiurak, 2013). Indeed, systematically varying the axis where these movements take place would prove valuable in order to assess the involvement of two different processes in these patients, that is, attention processes that may be at the basis of spatial stimulus-response associations, and intention processes that may generate response-effect associations.

## Conclusion

In conclusion, our results indicate that different action-related objects’ properties may activate multiple ways of interacting with them.

## Open Practices Statement

The data for all experiments are available at: https://osf.io/c8agd. None of the experiments was preregistered.

## Data Availability Statement

The raw data supporting the conclusions of this article will be made available by the authors, without undue reservation.

## Ethics Statement

The studies involving human participants were reviewed and approved by the Ethics Committee of the University of Bologna. The participants provided their written informed consent to participate in this study.

## Author Contributions

ES: conceptualization, methodology, data collection, data analyses, and writing – original draft preparation. SD’A: conceptualization, methodology, software, data analyses, and writing – reviewing and editing. LL and CI: conceptualization, writing – reviewing and editing, and supervision. SR: conceptualization and supervision. RN: conceptualization, methodology, and supervision. All authors contributed to the article and approved the submitted version.

## Conflict of Interest

The authors declare that the research was conducted in the absence of any commercial or financial relationships that could be construed as a potential conflict of interest.

## Footnotes

1 Note that the same sample size calculation was employed also for Experiment 2 where the same interaction was analyzed.

2
http://www.pstnet.com

3 Please note that to select and modify the new stimuli, the same method described in Experiment 1 was applied.

4 To rule out the possibility that the H-R compatibility effect for handle-only objects was affected by visual features (i.e., the material), a repeated measures ANOVA was conducted with *Handle-Response Compatibility* (compatible vs. incompatible) and *Material* (metal vs. plastic), as within-subjects’ factors. The interaction between Handle-Response Compatibility and Material was not significant, *F*(1,26) = 1.850, *p* = 0.185.
