# Corrigendum: Exploring the Role of Action Consequences in the Handle-Response Compatibility Effect

**DOI:** 10.3389/fnhum.2021.750105

**Published:** 2021-09-15

**Authors:** Elisa Scerrati, Stefania D'Ascenzo, Luisa Lugli, Cristina Iani, Sandro Rubichi, Roberto Nicoletti

**Affiliations:** ^1^Department of Education and Humanities, University of Modena and Reggio Emilia, Reggio Emilia, Italy; ^2^Department of Philosophy and Communication, University of Bologna, Bologna, Italy; ^3^Department of Surgery, Medicine, Dentistry and Morphological Sciences With Interest in Transplant, Oncology and Regenerative Medicine, University of Modena and Reggio Emilia, Modena, Italy; ^4^Center for Neuroscience and Neurotechnology, University of Modena and Reggio Emilia, Modena, Italy

**Keywords:** handle-response compatibility, response-effect compatibility, common coding of intention and action, ideomotor theory, affordance

In the original article, there was a mistake in ***Table 1*** as published. There was a **missing reference of Kourtis and Vingerhoets (****2015****)**. The corrected ***Table 1*** appears below.

**Table 1:** Prior known tests of the Handle–Response (H–R) compatibility effect showing null and/or reversed effects.

**Source, Study**	***N***	**Stimuli**	**Task**	**H-R compatibility effect**
Bub and Masson (2010)				
2	54	Beer mugs/Teapots	Color-judgement	Null
Cho and Proctor (2011)				
4	32	Door-handles	Color/Orientation judgement	Null
Cho and Proctor (2013)				
1	40	Door-handles	Shape-judgement	Null
6	20	Door-handles	Diagonal orientation judgement	Null
Kostov and Janyan (2012)				
1	37	Frying pans, Jugs, Saucepans	Orientation judgement	Null
Loach et al. (2008)				
2	20	Door-handles	Color-judgement	Null
Pellicano et al. (2020)				
1	20	Passive Torches	Length judgement	Null
2	20	Passive Torches	Length judgement	Null
3	20	Passive Torches	Size judgement	Null
Saccone et al. (2016)				
2	25	Mostly common elongated tools	Color-judgement	Null
Song et al. (2014)				
1	32	Passive Torches	Orientation judgement	Null
Symes et al. (2005)				
3	33	Mostly common elongated tools	Color-judgement	Null
Tipper et al. (2006)				
1	32	Door-handles	Color-judgement	Null
Yu et al. (2014)				
2A	30	Mostly common elongated tools	Orientation judgement	Null
2B	30	Mostly common elongated tools	Orientation judgement	Null
3A	30	Mostly common elongated tools	Orientation judgement	Null
Cho and Proctor (2011)				
1	64	Teapots	Color/Orientation judgement	Reversed
Cho and Proctor (2013)				
2	80	Door-handles	Color-judgement	Reversed
3	20	Door-handles	Color-judgement	Reversed
4	60	Door-handles	Color-judgement	Reversed
5	20	Door-handles	Color-judgement	Reversed
Loach et al. (2008)				
1	20	Door-handles	Texture judgement	Reversed
Kostov and Janyan (2015)				
2	58	Frying pans, saucepans bowls/plates	Orientation judgement	Reversed
3	51	Frying pans, saucepans bowls/plates	Orientation judgement	Reversed
Kourtis and Vingerhoets (2015)				
	29	Mostly common elongated tools	S-R compatibility	Reversed
Pellicano et al. (2010)				
1	20	Torches	Color judgement	Reversed
Pellicano et al. (2020)				
2	20	Active Torches	Length judgement	Reversed
3	20	Active Torches	Size judgement	Reversed
Proctor et al. (2017)				
1	20	Frying pans	Orientation judgement	Reversed
Yu et al. (2014)				
1A	32	Mostly common elongated tools	Artifact/Natural	Reversed
1B	32	Mostly common elongated tools	Artifact/Natural	Reversed
1C	32	Mostly common elongated tools	Artifact/Natural	Reversed

In the original article, there was an error. The authors mistakenly reported as evidence a hypothetical interpretation offered by Kourtis and Vingerhoets (2015) of their neurophysiological results. A correction has been made to *Introduction*, ***Paragraph*** **2**:

Evidence in favor of the H-R compatibility effect was initially provided by Tucker and Ellis (1998) who showed that judging the upright or inverted position of depicted graspable objects was influenced by the orientation of the object's handle. That is, responses were faster when the position of the handle and the responding hand were spatially aligned as compared to when they were not. This result was replicated across different tasks (e.g., Tipper et al., 2006; Saccone et al., 2016), stimuli (e.g., Pellicano et al., 2010; Pappas, 2014; Iani et al., 2018; Scerrati et al., 2019, 2020), populations (e.g., Dekker and Mareschal, 2013), response devices (e.g., Bub and Masson, 2010), and response modes (e.g., Phillips and Ward, 2002; Cho and Proctor, 2010; Proctor et al., 2017; Bub et al., 2018; for a review see Proctor and Miles, 2014; for a recent meta-analysis see Azaad et al., 2019).

The authors apologize for this error and state that this does not change the scientific conclusions of the article in any way. The original article has been updated.

## Publisher's Note

All claims expressed in this article are solely those of the authors and do not necessarily represent those of their affiliated organizations, or those of the publisher, the editors and the reviewers. Any product that may be evaluated in this article, or claim that may be made by its manufacturer, is not guaranteed or endorsed by the publisher.
